# Occult hepatitis B: Evolving challenges and new perspectives

**Published:** 2011-06-01

**Authors:** Mesut Akarsu, Funda Ugur Kantar, A Arzu Sayiner

**Affiliations:** 1 Dokuz Eylul University, Faculty of Medicine, Department of Gastroenterology, Izmir, Turke; 2 Dokuz Eylul University, Faculty of Medicine, Department of Clinical Microbiology, Izmir, Turkey

**Keywords:** Occult hepatitis B, Hepatitis B surface antigen

Dear Editor,

We read with great interest the article, "efficacy of hepatitis B vaccine in those who lost hepatitis B surface antigen during follow-up" by Taheri et al. published in Hepatitis Monthly [1]. In this article, the authors assessed the efficacy of HBV vaccine in those who lost their HBsAg without seroconverssion to anti-HBs antibody with no detectable anti-HBs antibody and HBV DNA in their sera. They reported that nearly 24% of chronic HBsAg-positive subjects who lost their HBsAg responded to HBV vaccine and the remaining cases need to be followed for occult HBV infection (OBI). By definition, OBI is the presence of HBV DNA in the liver tissue in HBsAg-negative individuals. In some cases, HBV DNA can be found in serum. OBI is mainly related to a strong suppression of viral activity in which host immune response and epigenetic factors play major role. As a consequence of this viral suppression, the amount of circulating HBV DNA is very low (usually < 200 IU/mL) or even undetectable in OBI cases, so the diagnosis of this condition requires a very sensitive HBV DNA PCR assay with a detection limit of < 10 copies of HBV DNA per reaction [2]. OBI status is significantly associated with presence of anti-HBV antibodies (anti-HBC and anti-HBs antibodies) but what must be remembered that more than 20% of cases with HBI are negative for all serum markers of HBV infection [3]. A number of explanations for the persistence of HBV-DNA in HBsAg-negative samples have been proposed, including integration of HBV DNA into host's chromosomes, mutations in the major hydrophilic loop of the S gene, the window period following acute HBV infection, underlying HCV co-infection, immunosuppression, poor ability of laboratory in detection of HBsAg and the presence of immune complexes in which HBsAg may be hidden [4].

As we all know, HBV infection is a serious global health problem, with two billion people infected worldwide and 360 million suffering from chronic HBV infection [5]. The prevalence of OBI among HBsAg-negative blood donors is quite variable depending on the level of HBV endemicity in the populations in different regions of the world. In Europe, OBI is detected in the range of 1:2000-1:20,000 donations collected [6]. Raimondo et al. analyzed liver tissue from patients without liver disease and showed a prevalence of 17% of OBI in these individuals [7]. In fact, OBI may be more prevalent than what we know today. The importance of OBI is its contribution to several different clinical contexts including the transmission of HBV infection, the risk of reactivation, the contribution to liver disease progression and to the development of hepatocellular carcinoma (HCC). There is a general agreement that HBsAg-negative blood donations containing viral DNA have to be considered as infectious and may transmit HBV during blood transfusions.

In routine practice, cases with positive anti-HBc but negative HBsAg and anti-HBs are not uncommon, especially in regions where the prevalence of hepatitis B is high. There are several published studies related to hepatitis B vaccination in isolated anti-HBc-positive cases. In a study from Korea, the first and third vaccinations caused successful immunity in 70.6% and 70.6% in isolated anti-HBc group, and 34.4% and 81.2% in the control group, respectively [8]. A recent study from Turkey, showed that the response to hepatitis B vaccination in persistent isolated anti-HBc-positive subjects was 50%, 68.7% and 89.6% after the first, second and third vaccination, respectively [9]. HBsAg may be undetectable due to very low levels of the antigen or escape mutations. Selection of a sensitive HBsAg assay which can detect S gene mutants is very critical to identify the infection. Evaluation of 17 CE-marked HBsAg assays showed that analytical sensitivities of the assays ranged from 0.009 to 0.05 PEI-U/mL for HBsAg "ad" standard and 0.012 to 0.11 PEI-U/mL for the "ay" standard. In addition, detection of HBsAg mutants was problematic with several assays [10]. Similarly, detection of anti-HBs is also problematic due to differences among the commercial assays. Heijting et al. showed that there is a vaccine-dependant discrepancy among some commercial anti-HBs assays and that determination of anti-HBs level depends on the test and the vaccine [11]. Testing the samples with a different HBsAg and anti-HBs assay could give a different result and help to solve the undetermined cases reported in the study of Taheri et al.

The occurrence of anti-HBs antibody response indicates viral clearance although there are rare exceptions. It would be wise to vaccinate cases with isolated anti-HBc and negative HBV DNA to decide whether the patient has OBI or resolved infection since anti-HBs response to HBV vaccine in such individuals is generally accepted as the proof of resolved infection [12]. Taheri et al. preferred to test for antibody response after three doses of vaccine as this was the schedule for the control group. Even after the third dose, nearly 77% of the cases were non-responders which raised the question whether these patients have OBI. The answer requires either liver biopsy or long-term follow-up as mentioned by the authors of the article. Another question about this issue is whether the vaccine protects against escape mutants. In a recent study 3.7 million blood donations were tested with nucleic acid testing (NAT) and nine HBV DNA-positive but HBsAg- and anti-HBc-negative donations were found. Surprisingly, six of the nine donors have been vaccinated and had low levels of anti-HBs (3-100 IU/L). When analyzed, HBV strains isolated from three of the vaccinated donors were essentially wild type whereas quasi-species and escape mutations were found in other three donors. It is concluded that low anti-HBs levels induced by vaccination protect against hepatitis B disease and chronic infection but favor OBI [13]. What must be done is to control anti-HBs titers in vaccinated persons and to remain the titers > 100 IU/L. Generation of new hepatitis B vaccines which are protective against to escape mutants might be the solution.
